# State-of-the-Art Research on “Lymphomas: Role of Molecular Imaging for Staging, Prognostic Evaluation, and Treatment Response”

**DOI:** 10.3389/fonc.2013.00212

**Published:** 2013-09-04

**Authors:** Lale Kostakoglu, Bruce D. Cheson

**Affiliations:** ^1^Department of Radiology, Mount Sinai Medical Center, New York, NY, USA; ^2^Division of Hematology-Oncology, Lombardi Comprehensive Cancer Center, Georgetown University Hospital, Washington, DC, USA

**Keywords:** PET/CT, Hodgkin lymphoma, non-Hodgkin lymphoma, interim-PET, response surrogate

## Abstract

Lymphomas are heterogeneous but potentially curable group of neoplasms. Treatment of lymphomas has rapidly evolved overtime with significant improvement in the cure rate and reductions in treatment-related toxicities. Despite excellent results, treatment programs are continued to be developed to achieve better curative and safety profiles. In these patients individualized therapy schemes can be devised based on a well-defined risk categorization. The therapy efficacy can be increased early during therapy in non-responding patients with escalated therapy protocols or with the addition of radiation therapy, particularly, in advanced-stage or unfavorable risk patients. The increasing availability of positron emission tomography using 18F-fluorodeoxyglucose, particularly fused with computed tomography (FDG-PET/CT) has lead to the integration of this modality into the routine staging and restaging for lymphoma with convincing evidence that it is a more accurate imaging modality compared with conventional imaging techniques. FDG-PET/CT is also is a promising surrogate for tumor chemosensitivity early during therapy. This review will summarize published data on the utility of FDG-PET/CT imaging in the staging, restaging, and predicting therapy response in patients with lymphoma.

## Introduction

The lymphomas are a heterogeneous group of diseases with respect to their biology, treatment, and prognosis. Despite the high rate of cure of Hodgkin lymphoma (HL) and diffuse large B-cell lymphoma (DLBCL), there is a need to alter therapy in patients unlikely to benefit from standard treatment, while reducing treatment intensity in patients with low risk disease. To achieve this goal requires not only an accurate staging system, but strong baseline risk factors (prognostic), and/or those early during therapy (predictive factors) to define the optimal treatment strategy. Positron emission tomography (PET) using F-18-fluorodeoxyglucose (FDG), integrated with computed tomography (CT) (PET/CT) has become widely used in the staging and evaluation of therapy response in lymphomas, and may provide the means for such an individualized approach.

### Histopathologic lymphoma subtypes

F-18-fluorodeoxyglucose-avidity varies among the various lymphoma subtypes, the most routinely avid being HL, DLBCL, Burkitt, mantle cell (MCL), follicular lymphoma (FL) with a PET/CT sensitivity of 85–100% (1–6). There is currently no established role for the clinical usefulness of FDG-PET/CT in the less common indolent NHLs including marginal zone lymphomas (MZL), chronic lymphocytic leukemia/small lymphocytic lymphoma (CLL/SLL), and lymphoplasmacytic lymphoma (LPL), that tend to have limited and variable FDG-avidity (3, 7–12). About 10% of NHLs are of T-cell origin (13) and FDG-PET avidity is variable; being most reliable for the more aggressive, nodal subtypes including peripheral T-cell lymphoma not otherwise specified and anaplastic large cell lymphoma (12, 13).

F-18-fluorodeoxyglucose-avidity appears to correlate with aggressiveness of lymphomas (14, 15) and imaging with FDG-PET may be helpful in identifying a potential site of histologic transformation. It is important to note that SUVs exceeding 10 yields 80% certainty for the identification of aggressive behavior (16, 17), particularly, in Richter’s transformation for patients with CLL/SLL (18).

## Staging of Lymphomas

Ann Arbor staging, the most widely used system, has evolved over the past 40 years to incorporate CT (19, 20). Nevertheless, anatomic imaging relies on size and location and, therefore, is unable to distinguish malignant from benign lymph nodes (21). Numerous studies and a meta-analysis demonstrate that FDG-PET is more accurate than CT at initial staging with a maximum joint sensitivity and specificity of 96% on a lesion basis (22), which far exceeded the corresponding values for contrast-enhanced computed tomography (CECT) (2, 9, 22–26). Discordance between PET and CECT findings occurs in approximately one third of patients at initial staging, predominantly, in favor of PET/CT imaging (22, 24, 26–32); however, stage is uncommonly altered (in up to 30% of patients), and treatment is rarely changed (in up to 15% of patients) with no evidence that outcome is improved as a result of these data (2, 24–29, 36–42). It is important to realize that the widespread use of systemic chemotherapy in lymphoma patients appears to mitigate the need for a precise determination of the anatomic extent of disease; however, staging PET/CT is integral to evaluation of subsequent response to therapy.

The International Harmonization Project (IHP) recommends a baseline FDG-PET scan for HL and DLBCL because of their consistent FDG-avidity and potential curability (33). For other subtypes, FDG-PET imaging is recommended for clinical trials, particularly, when response rate is the primary objective. The current National Comprehensive Cancer Network guidelines (NCCN) recommend baseline PET imaging as an essential test in HL, DLBCL, AIDS related B-cell lymphomas and as a useful test in selected cases in FL, MZL, MCL, but does not recommend it in CLL/SLL (34, 35).

### PET plus CECT at staging

Whether the addition of a CECT improves the sensitivity and specificity of PET/CT remains controversial (24, 25, 32, 41–46). In a series of 103 NHL and HL patients, Raanani et al. reported that the addition of CECT to PET/CT changed management in only about 10% of patients while FDG/PET resulted in a management change in almost 50% of HL patients compared with CECT alone (41). However this study described neither what type of treatment changes occurred nor whether outcome was altered. In a group of 47 NHL or HL patients, Rodríguez-Vigil et al. reported no significant differences between the use of unenhanced low-dose PET/CT and contrast-enhanced full-dose PET/CT, although PET/CECT produced fewer indeterminate findings and identified a higher number of extranodal sites (42). These results suggest a marginal benefit with the addition of CECT to PET examination. In current practice, management of lymphoma usually requires both CECT and low-dose FDG-PET/CT for morphologic and metabolic assessment, respectively. However, this strategy increases patient radiation exposure by up to twofold (45).

Contrast-enhanced computed tomography is advisable in patients with abdominal lymphomas for distinguishing lymph nodes from non-opacified bowel loops and vessels, and where more precise measuring of node size is indicated. In rare cases with head and neck involvement, CECT may be useful to differentiate physiologic uptake from enlarged cervical lymph nodes. While in lymphomas with variable and low-grade FDG uptake including CLL/SLL and MZL, and some PTCL (3, 8, 14, 47, 48), CECT should be the imaging modality of choice. Further consideration for the use of both FDG-PET and CECT includes patients who are planned to undergo radiation therapy.

### FDG-PET in bone marrow involvement

Accurate assessment of the BM is crucial because it often upstages disease, leading to alterations in therapy strategy (5, 6). Lymphoma involvement of the BM is more common in patients with NHL (20–30%) (49, 50), especially, in indolent subtypes and MCL compared to those with HL (10%). BM biopsy is known to have a substantial false-negative rate due to the small volume of samples (51), and that it does not evaluate marrow involvement outside the pelvis. The sensitivity of PET in detecting BM involvement in HL and NHL, primarily in DLBCL, is about 90 and 75%, respectively (52–59) while only a sensitivity of 50% was achieved in indolent NHL (52, 53). The lower sensitivity seen in DLBCL can be explained by discordant lymphoid infiltrates representing the low-grade component of disease that lowers the sensitivity of FDG-PET imaging (49, 50). Although BMB remains essential for the diagnostic work-up only rarely do the early stage HL patients have BM involvement (58–60). BMB should no longer be recommended for staging clinically advanced HL because the marrow is virtually never involved in the absence of constitutional symptoms or other evidence of stage IV disease (60). Consequently, routine BMB should be restricted to patients with NHL. Patients with DLBCL rarely have a positive bone marrow biopsy in the absence of focal or diffuse involvement by PET-CT, or who have other evidence of advanced disease and, therefore, this procedure should be restricted to those with a positive scan to assess for the presence of a discordant histology. Even in indicated patients FDG-PET should precede BMB and biopsy or MRI could be pursued for confirmation of a positive PET finding. In the post-therapy setting, one should be mindful of the reactive BM changes induced by the colony stimulating factors (i.e., G-CSF). A 4–6 week period of time should be allowed before post-therapy PET imaging in patients who have received G-CSF to minimize the risk of a false-positive interpretation of the BM.

## Evaluation of Response after Completion of Therapy

Perhaps the clearest role for the use of PET in lymphoma is in post-treatment response assessment because of its ability to distinguish fibrosis or sclerosis from residual active disease. Early studies have demonstrated a role for post-therapy FDG-PET imaging in the prediction of aggressive NHL or HL recurrence (61–69). A negative (NPV) and a positive predictive value (PPV) of 80 and 100% were reported for FDG-PET in the identification of residual aggressive NHL after completion of first-line chemotherapy (66). In HL patients, studies demonstrate significantly shorter progression-free survivals (PFS) for PET-positive patients (0–4%) compared with 85–95% for those with a negative scan (62, 63, 68, 69). The revised International Working Group response criteria (rIWG) incorporated FDG-PET to accurately assess post-therapy persistent masses in both NHL and HL (33). The rIWG-PET interpretation criteria (i.e., IHP criteria) eliminated the terminology of “complete remission/unconfirmed (CRu)” on the basis of better response characterization provided by FDG-PET imaging. However, there is a need to prospectively validate these criteria in HL and DLBCL after first-line therapy as the majority of prior studies using IHP were based on retrospective data.

### Indolent lymphomas

Limited FDG-PET data exist for FDG-PET in low-grade NHL (7, 70–74). In 45 untreated FL patients, Le Dortz et al. reported a median PFS of 48 and 17.2 months in the PET/CT-negative and positive groups, respectively, after four or six cycles of induction immunochemotherapy (7). Similar results were obtained by Bishu et al. in a retrospective review of 31 FL patients treated mainly with R-CHOP therapy (70). In another series of 39 relapsed or refractory FL patients, after completion of bendamustine therapy, the percent reduction in SUVmax (70 vs. 29%) and in maximum perpendicular diameters (78 vs. 48%) were significantly greater in patients achieving a CR than in those with non-CR (71). The use of PET at the end-of-treatment in high-tumor-burden FL is supported by the emerging data. The utility of FDG-PET/CT in assessing response at the end of induction immunochemotherapy was suggested by the Primary Rituximab and Maintenance (PRIMA) study by the GELA in high-tumor-burden FL patients (72, 73). Patients remaining PET-positive had a significantly inferior PFS at 42 months than in those who became PET-negative (33 vs. 71%, *p* < 0.001) in a subgroup of 122 patients (73). Similarly in a prospective study of 121 previously untreated high-tumor-burden FL patients at the end-of-treatment (first-line immunochemotherapy with six cycles of R-CHOP plus two cycles of rituximab, without rituximab maintenance) (74). When the response was assessed using Deauville criteria, with a median follow-up of 23 months, 2 year PFS was 87% for final PET-negative vs. 51% for final PET-positive patients (*p* < 0.001), respectively. End-of-treatment, but not interim scans, were predictive of 2 year OS for positive and negative scans (*p* = 0.013).

### Stem cell transplantation

A current standard treatment for relapsed or refractory HL involves high-dose chemotherapy and autologous hematopoietic stem cell transplantation (HDT/ASCT), offering long-term disease free survival in more than 50% of transplanted patients (75). Favorable outcome is largely a function of chemosensitivity at the time of ASCT (76–84). In recent studies in HL, best ASCT response was obtained in patients with chemosensitive disease who were PET-negative (76–80) after salvage therapy regardless of the chemotherapy that induced the response (79). A recent meta-analysis from 12 studies with 630 patients (187 HL; 313 DLBCL) reported a sensitivity of 69% and specificity of 81% (83). Additionally, PET-positive disease was associated with a significantly inferior 3 year PFS or EFS (31–41%) compared with patients who had PET-negative results following salvage chemotherapy prior to ASCT (75–82%) (76–78). Similar results were obtained in a retrospective case-series of 39 primary refractory or relapsed DLBCL patients with 3 year PFS of 35 vs. 81% for patients with positive pre-ASCT PET vs. those who had a negative PET (*p* = 0.003) (85). Consequently, post-salvage therapy FDG is recommended to differentiate patients with a better prognosis following ASCT from others with unfavorable prognosis.

## Response Evaluation during Therapy

Rapid response to chemotherapy is a recognized surrogate marker of chemosensitivity in both HL and DLBCL with an attendant high likelihood of a longer PFS (86). Persistent FDG uptake after two to four cycles of chemotherapy is associated with relapse rates ranging from 50 to 100%, while the relapse rate in interim-PET-negative patients is usually lower than 10% (87–92). In a meta-analysis, interim FDG-PET yielded an overall sensitivity of 81% and a specificity of 97% for advanced-stage HL, and a sensitivity of 78% and a specificity of 87% for DLBCL (93). Nonetheless, more recently, the results obtained for DLBCL patients were less convincing (94).

In advanced-stage HL patients (*n* = 260), after two cycles (PET2) of standard therapy, Gallamini et al. reported treatment failure in 86% of PET2-positive patients after a median follow-up of 2.2 years while 95% of PET2-negative patients remained in CR (88). Interim-PET results have also been shown to be a stronger predictive factor for PFS than the International Prognostic Score (IPS) (88, 91). Similarly, in another non-randomized prospective study of mixed stage HL patients, Cerci et al. reported a 3-year EFS of 55 and 94% for PET2-positive and negative patients, respectively (*p* < 0.001) (95). However, these data should also be interpreted with caution because IPS categories were not restricted to advanced-stage patients and unfavorable factors were disregarded in the stage classification.

The role of FDG-PET in the prediction of ultimate outcome is clearer in advanced-stage than early stage HL (93–103). Hutchings et al. reported a PPV of only 30% for an interim-PET after two or three cycles of ABVD chemotherapy in early stage HL (90, 91) while the NPV was high at 95%. More recently, in limited stage non-bulky HL patients the enthusiasm for interim-PET imaging has been tempered with no clear difference noted between PET2-positive and negative patients with respect to PFS (87 vs. 91%; *p* = 0.57) (98). By contrast, end-chemotherapy PET was highly predictive of PFS (94 vs. 54%; *p* < 0.0001). However, these are retrospective data with no control imposed over PET acquisition protocols and standardization of timing which are essential factors to provide reliability and reproducibility for the results.

Another important consideration is that the effectiveness of therapy has an influence on the predictive value of any given predictive marker. Using a slightly less effective chemotherapy regimen (doxorubicin, vinblastine, and gemcitabine), in a non-randomized prospective study of early non-bulky HL, Straus et al. reported a lower than expected 2 year PFS at 88% for PET2 negative patients while the PFS was 54% in the PET2-positive group (*p* = 0.0009), (96, 97). Similarly, in a randomized, prospective trial by Le Roux et al. early and advanced-stage HL patients were treated with a therapeutic strategy adapted to baseline prognostic factors, interim-PET after four cycles of ABVD (PET4) and CECT (99). The negative NPV and PPV for the interim FDG-PET predicting 2 year PFS were 96 and 16%, respectively (*p* < 0.0001). The inferior PPV obtained in this study is not surprising as treatment intensification schemes may negate the predictive value of PET positivity.

Based on compelling data on interim-PET in advanced-stage HL, multiple PET-directed randomized studies were initiated to determine the outcome of therapy escalation in non-responding patients as well as de-escalation in patients who achieve an early CR. But, only few have reported interim results (100–104). In the HD15 trial of the German Hodgkin Study Group (GHSG), HL patients (stages IIB, III, IV) were initially randomized to one of three induction regimens (102). Those with a residual mass of at least 2.5 cm underwent a PET scan. Patients with a negative study were not further treated, whereas those with a positive scan received involved field radiation. The frequency of consolidative IFRT was only 11% compared with 70% in prior studies antedating the use of PET scans, with no difference in overall survival. In a retrospective analysis of a prospective study by Gallamini et al. in advanced-stage HL (GITIL/HD0607) when the treatment of PET2-positive patients was escalated to BEACOPP (bleomycin, etoposide, adriamycin, cyclophosphamide, vincristine, procarbazine, and prednisone) regimen the FFS was 95% in PET2 negative and 62% in PET2-positive groups (*p* < 0.0001) with a median follow-up of 34 months which was superior to the 15% in patients whose therapy was unchanged (103). Another adaptive therapy trial in advanced-stage HL by Dann et al. using not only interim-PET results but also the IPS for stratifying patients into different therapy arms reported a 10-year PFS of 83% in interim-PET-positive patients compared with 93% for those with a negative interim-PET (ns) suggesting that unfavorable outcomes can be overcome by therapy intensification (100).

In the RAPID trial, patients with limited stage disease received three cycles of ABVD (104). Those who were PET-positive received an additional cycle followed by radiation therapy. The negative patients were randomized to involved field RT or observation, the latter being shown to be non-inferior. These results support the use of PET in risk-adapted strategies.

In DLBCL, while FDG-PET at completion of therapy is a good predictor of outcome, the value of an interim-PET remains controversial because of its low PPV (93). Although earlier studies supported a role for an interim FDG-PET performed after two to four cycles of standard chemotherapy, the results of these and later studies varied significantly among patient groups (105–107). The 2-year PFS for the PET-negative groups was 82–93% in PET-negative while the PFS for the PET-positive groups varied from 0 to 43%. These differences in PET results may be related to varying follow-up periods, patient populations, and different types of treatments employed, i.e., standard chemotherapy alone or with immunotherapy (rituximab). To further clarify the clinical relevance of interim FDG-PET, in a risk-adapted dose-dense immunochemotherapy program, Moskowitz et al. reported similar PFS for interim-PET-positive/biopsy-negative patients and in interim-PET-negative patients during a follow-up of 44 months (94). In PET-positive patients, repeat biopsy was negative in 87%, and 51% of these patients remained progression-free after consolidation therapy during follow-up.

To date, the majority of interim FDG-PET studies have used visual criteria. However, the results of the GELA trial (LNH2007-3B) of 85 high-risk DLBCL patients suggested that those patients whose tumors had a percent SUV change (ΔSUVmax) of >66% between baseline and after two cycles of therapy (2 year PFS 77 vs. 57%; *p* = 0.028) and >70% between baseline and after four cycles (2 year PFS 83 vs. 40%; *p* < 0.0001) could be spared high-dose therapy (108). On the contrary, outcomes did not differ significantly whether PET2 and PET4 were visually positive or negative. The GAINED trial by the same investigators is designed to further demonstrate the superiority of quantitative approach over visual interpretation (109).

In summary, in advanced-stage HL, encouraging data exist on the effectiveness of interim FDG-PET/CT as a surrogate for chemosensitivity. However, there is limited evidence that changing treatment based solely on interim-PET-CT results improves patient outcome. Interim-PET-adapted therapy strategies should be pursued only in a clinical trial setting until the value of interim-PET is proven by ongoing prospective, “response-adapted” therapy trials. The role of FDG-PET in DLBCL and early stage HL are not supported by the available data. Furthermore, there is no evidence to suggest that an early therapy change in the poorly responding patients will translate into a survival benefit.

## Surveillance Following First-Line Therapy

Despite improvements in survival rates, relapses occur in approximately 30–50% of advanced-stage HL and DLBCL patients following first-line therapy (110–112). In a meta-analysis, the sensitivity and specificity of FDG-PET in identifying disease relapse for HL were 50–100 and 67–100%, respectively, and for NHL 33–77 and 82–100%, respectively, irrespective of a residual mass on CT (113). In a recent study of HL and aggressive NHL, more than 60% of relapses were diagnosed clinically, especially, in aggressive NHL and in cases with extranodal involvement. Although HL relapses were more commonly detected by FDG-PET scans because of clinically silent disease, no survival benefit was proven (114). In another study of 421 patients with mixed histologies including HL, aggressive NHL, and FL after first complete remission, serial six monthly FDG-PET scans enabled detection of relapse within 18 months of therapy (115). There are also conflicting results reporting a PPV of only 30% for FDG-PET in HL patients (116).

In summary, survival does not appear to be affected by mode of detection of recurrent lymphoma or the frequency of imaging. The low PPV associated with follow-up FDG-PET scans negates their clinical value in identifying patients who would benefit from additional treatment (117, 118).

## General Considerations and Recommendations

### Timing of FDG-PET imaging

- Interim-PET should be scheduled within 4–5 days of start of the subsequent therapy cycle to minimize false-positive results produced by the florid inflammatory response that peaks at around day 10 of chemotherapy initiation (118, 119).
- The timing of FDG-PET studies after chemotherapy completion is more flexible, a 6- to 8-week window after end of therapy should be observed to allow for inflammation to subside and to minimize false-positive results caused by the inflammatory response associated with rituximab therapy (120).
- Although, the bulk of existing data supports the use of interim-PET after two cycles of treatment in HL, there is no established optimal timing with regards to therapy cycles. If and when a paradigm shift toward tailored approach is established, performing PET after two cycles seems reasonable. There is also evidence that PET after one cycle has a high negative predictive value with respect to PFS (121, 122).

### Standardization of FDG-PET interpretation

- The International Harmonization Project criteria were developed for evaluation of response after completion of therapy. IHP criteria use the mediastinal blood pool as an internal reference for lesions of 2.0 cm or larger to discriminate a positive finding from a negative (118).
- To increase the specificity of PET readings, the definition of a positive interim-PET result has evolved from any uptake above background to uptake intensity that is equal to the mediastinal blood pool, i.e., IHP criteria (118), and more recently to an intensity exceeding the background in the liver (122, 123).
- For interim-PET readings, a relatively high cut off is appropriate to measure chemosensitivity. Recently proposed “Deauville criteria” yield a flexible reading scheme suitable for different positivity thresholds to adjust for the intended treatment end-points (122, 123).
- Deauville criteria have recently been validated in a retrospective cohort of 260 advanced-stage HL patients treated with ABVD (124). After a mean follow-up of 27.2 months, the 3-year PFS of PET2-positive and negative patients were 28 and 95%, respectively (*p* < 0.001). The binary concordance between paired reviewers was high (k Cohen: 0.84).
- The widely recognized challenge to the integration of interim-PET into management schemes is the variability and the high false-positive rates associated with visual evaluation, particularly in those with bulky residual masses.

### Semi-quantitative evaluation

- Metabolic changes determined by the SUV which is adjusted for body weight and administered activity, provide a continuous and objective scheme of measurements that are more compatible with the kinetics of *in vivo* therapy response.
- The change in tumor SUVmax before and after treatment can be used as a measure of response. However, strict adherence to protocols for all imaging periods are necessary because SUV measurements depend on multiple variables including time interval after injection, blood glucose level, body weight, and technical PET parameters.
- The use of quantitation to improve upon visual assessment was explored in DLBCL after two and four cycles of chemotherapy by Casasnovas et al. (108, 125). The results of this study have been previously discussed in Section “Early Response Evaluation during Therapy.”
- Further studies are needed to define a widely accepted semi-quantitative approach for lymphoma, probably with slightly different values for each subtype.

### Future directions in quantitative PET assessments

- Disease bulk at initial presentation has long been a known adverse prognostic factor, particularly in early stage HL (126). Several methods can be used to measure disease bulk, including the mediastinal-thoracic ratio and maximum size of the largest mass. However, studies are underway to evaluate PET-based metabolic tumor volume (MTV) or total lesion glycolysis (TLG) as more accurate methods to determine disease burden by accounting for the whole body tumor volume using sophisticated software systems (127–130).
- Preliminary MTV data are available for patients with DLBCL (131, 132). In 169 stage II-III DLBCL patients treated with R-CHOP, multivariate analysis revealed an association between high MTV group and lower PFS and OS during a median follow-up of 36 months (*p* < 0.001), but not with stage III (*p* = 0.054) (131). These results suggest a higher predictive power for MTV compared to Ann Arbor staging in DLBCL patients.
- The prognostic value of these automated volumetric methods will be determined after the establishment of the optimal method for determining the most accurate tumor volume.

## Conflict of Interest Statement

The authors declare that the research was conducted in the absence of any commercial or financial relationships that could be construed as a potential conflict of interest.
